# Comparative Analysis of the Measles Antibody Levels in Healthy Medical Personnel of Maternity Ward and Women in Labor

**DOI:** 10.3389/fimmu.2021.680506

**Published:** 2021-07-08

**Authors:** Mikhail Petrovich Kostinov, Pavel Ivanovich Zhuravlev, Lylia Solomonovna Gladkova, Kirill Vadimovich Mashilov, Valentina Borisovna Polishchuk, Anna Dmitrievna Shmitko, Veronika Nikolaevna Zorina, Dmitriy Alexeyevich Blagovidov, Dmitriy Vladimirovich Pahomov, Anna Egorovna Vlasenko, Alexey Anatolevich Ryzhov, Ekaterina Alexandrovna Khromova

**Affiliations:** ^1^ Department of Epidemiology and Modern Vaccination Technologies of I.M. Sechenov First Moscow State Medical University, Moscow, Russia; ^2^ Laboratory of Vaccine Prophylaxis and Immunotherapy of Allergic Diseases of I.I.Mechnikov Research Institute of Vaccines and Sera, Moscow, Russia; ^3^ City D.D. Pletnev Clinical Hospital of the Moscow City Health Department, Moscow, Russia; ^4^ Department of Epidemiology and Social Hygiene of Moscow State University of Food Industries, Moscow, Russia; ^5^ Deputy Director for Science of Institute of Highly Pure Biopreparations, Saint Petersburg, Russia; ^6^ Medical Cybernetics and Informatics Department of Novokuznetsk State Institute of Advanced Training of Physicians—Branch of the “Russian Medical Academy of Continuous Professional Education” of the Ministry of Healthcare of the Russian Federation, Novokuznetsk, Russia

**Keywords:** measles prevention, maternity ward, women in labor, seropositivity, measles IgG antibodies

## Abstract

It has been proven that post-vaccination immunity to measles virus after two doses of vaccine is not able to persistently protect against infection throughout life. The goal of this research was to determine the immune layer to the measles virus among women in labor and maternity ward personnel in the same medical institution. The levels of IgG antibodies to measles virus in the umbilical cord blood of 594 women in labor and 88 workers of the maternity ward were studied by ELISA. It was revealed that 22.7% of umbilical cord blood serum samples from parturient women and 21.4% of blood serum samples from maternity ward personnel were seronegative (<0.18 IU/ml). Levels of IgG antibodies to measles virus in low values (<1.0 IU/ml) were detected in 67% of blood serum samples among women in labor and 68.9% among employees of the maternity ward. Among women in labor, women under 35 years of age are at the highest risk of contracting measles; the proportion of women with low levels of protective antibodies in this age group was almost 70%, and the proportion of women without protective levels of antibodies was 23%. Compared with the age group 36–43, the age of women in labor under 35 was associated with a higher chance of not having immune protection against infection with measles virus OR [95% CI] = 2.2 [1.1–4.5] (p = 0.02) or had a low level of protection OR [95% CI] = 1.9 [1.2–3.0] (p = 0.001). It was also found that among women over 35 years of age, the proportion of persons with a high level of antibodies in women in labor was statistically significantly higher than among members of the maternity ward staff (13 and 0%, respectively, p = 0.007). Thus, maternity ward employees and women in labor constitute a risk group for measles due to the presence of a high proportion of seronegative persons among women of childbearing age (both maternity ward employees and women in labor). These conditions create the need to revise current approaches to present vaccination procedures, especially in the current epidemiological situation with COVID-19.

## Introduction

Measles is an extremely highly contagious disease with a reproduction index of 12 < R_0_ < 18 caused by a virus, which quite often leads to serious complications and even death. Although the widespread use of the measles vaccine has led to a sharp decrease in the incidence, according to the WHO, up to 30 million measles cases are annually registered in the world, of which about 50 thousand are fatal, and after steady global progress from 2010 to 2016, the number of reported measles cases climbed progressively to 2019 (1, 2).

Globally, against the background of a general trend towards a decrease in the incidence of measles (by 84% from 2000 to 2016), at the end of 2017, 109,638 deaths from measles were registered; this is almost 20,000 additional deaths compared to 2016 (an increase of 22%) (3). After a major measles epidemic in 2017, with 4,347 confirmed cases, 2,029 measles cases were reported by the Italian national measles and rubella surveillance system in the first half of 2018 (4). Although measles eradication was officially announced in the United States in 2000, measles outbreaks were increasing, with 1,018 hospitalizations reported in the United States between 2002 and 2016, and the number of hospitalizations increased over time (5).

This trend is associated with a decrease in the level of population immunity. Although it is generally accepted that the live attenuated measles vaccine is highly effective, and especially two doses of the vaccine guarantee protection against the disease in 94.1% of cases, the intensity and duration of post-vaccination immunity may be lower compared to the level of immunity acquired after an infection with a wild-type virus, since annually 15–20% of cases fall on vaccinated and revaccinated persons, and in some years their share is up to 30% (6, 7). When examining more than 1,000 children in Canada, it was revealed that the titer of measles antibodies in them falls by an average of 5.6%, that is, the intensity of measles immunity will be almost halved over 12 years (8). It is known that HIV-1 and some other conditions associated with immunosuppression (for example, transplantation and primary immunodeficiency) also lead to impaired development and/or weakening of immunity caused by the use of the measles vaccine (9). A year after the revaccination of children with allergic reactions or frequent respiratory infections, the proportion of seronegative patients can reach 30% (10).

At the present stage, the highest incidence rates are recorded in the age group of children under 1 year old, and the largest share in the older age groups is made up of persons aged 25–39 years (5). Thus, persons with the highest reproductive potential are more likely to become infected with measles, as they lose post-vaccination antibodies over time, and children born to mothers without protective antibody levels remain susceptible to the measles virus until the first dose of vaccine is given at 12 months of age.

Measles is especially dangerous during pregnancy since it is characterized by a severe course and high mortality due to complications such as pneumonia and respiratory failure. These features are accompanied by an increased risk of premature birth and the development of placental insufficiency.

Against the background of the high incidence of measles, the emergence of an outbreak of measles infection in maternity hospitals is of deep concern. In such cases, doctors and healthcare professionals are at increased risk. According to the results of the analysis of 1,053 medical workers in Turkey, the seropositivity rate for measles among them was 57.1%, the levels of susceptibility to measles among other study participants in different age groups—18–26, 27–38, and over 38 years old—were statistically significantly different (46, 18.6%, and 0%, respectively; p <0.001) (11). When France faced a severe measles outbreak in 2010–2011, healthcare workers accounted for 9% of patients (12).

In addition, at present, in connection with the COVID-19 pandemic, the problem of the intensity of measles immunity acquires a new meaning, since there are currently several directly and indirectly confirmed hypotheses about its possible role in preventing the spread of SARS-Cov-2 infection and reducing mortality.

The study aimed to analyze the intensity of measles immunity based on the content of anti-measles IgG antibodies in maternity ward personnel and women in labor.

## Methods

The study was carried out as part of a program to improve measles prevention and analysis of the reasons for the increase in the proportion of seronegative persons.

### Description of Groups

The study was taken at the clinic of the Moscow Regional Research Institute of Obstetrics and Gynecology in Moscow from November 2011 to June 2012. The samples that were analyzed included 594 samples of umbilical cord blood serum taken from women in labor who had no previous measles and 88 samples of venous blood serum taken from employees of the maternity ward of the D.D. Pletnev City Clinical Hospital of Moscow City Health Department at the same period.

Blood samples were taken during working hours, in the morning, in compliance with the rules of antiseptics and ethical standards. All study participants signed informed consent to participate in the study.

The following inclusion criteria were defined for the study groups:

 1. female;
 2. known vaccination history;
 3. availability of documentary evidence that confirmed vaccination and revaccination against measles;
 4. age from 21 to 43 years old;
 5. no acute respiratory illness at the time of the study;
 6. having informed consent to participate in the study.

Since the production of post-vaccination antibodies and the duration of their retention can be influenced by various factors and health conditions of the respondents, the following exclusion criteria were determined:

 1. current therapy with immunosuppressive drugs, systemic administration or inhalation of high doses of corticosteroids (more than 800 mcg per day of beclomethasone or equivalent), radiation therapy, cytotoxic drugs, or non-steroidal anti-inflammatory drugs;
 2. HIV infection (positive serological test), hepatitis B (acute) and hepatitis C (acute);
 3. therapy with immunoglobulins and other products of processing of donated blood within 90 days before participation in the study;
 4. administration of vaccines within 30 days of enrollment in the study;
 5. having chronic diseases.

The vaccination history of the employees was checked against their medical records; the vaccination history of the women in labor was collected by questionnaires. All persons included in the study stated that measles vaccination was carried out according to the vaccination schedule (two times) and confirm that there were no cases of measles or contact with patients with measles.

### Laboratory Methods

The level of anti-measles IgG in blood serum samples was detected by ELISA using the Vector-Best IgG-measles test system (Russia) according to the attached manufacturer’s instructions. According to the attached normative and technical documentation for the quantitative determination of IgG antibodies to measles virus, the test result was considered negative if the concentration of antibodies in the test sample was less than 0.11 IU/ml; positive if greater than or equal to 0.18 IU/ml. Sera with a borderline concentration of IgG antibodies to measles virus in the range of 0.12–0.17 IU/ml were assigned as negative values since these levels of antibodies cannot be considered as completely protective against the development of the disease. The positive levels of antibodies to measles were conventionally divided into three categories: low—less than 1.0 IU/ml, medium—from 1.0 to 5.0 IU/ml, and high—more than 5.0 IU/ml.

### Statistical Analysis

Comparison of the age between seronegative and seropositive women was performed by the Mann–Whitney method. Comparison of proportion was carried out using the Chi-Square test (Fisher’s exact test as need). Descriptive statistics of quantitative data are presented by the median and interquantile range and quantitative statistics by the share of persons with the attribute in question in the group, indicating the 95% confidence interval calculated by the Clopper–Pearson method. Youden’s method was used to find the cutoff point. The odds ratio was calculated to determine the association between the age group and IgG level. All calculations were performed in the free software environment for computing “R” (“The R Project for Statistical Computing”) (v.3.6.0).

## Results

A study of umbilical cord blood serum samples from women in labor for the presence of IgG antibodies to the measles virus showed that more than 20% of the women in labor were seronegative (<0.18 IU/ml) (**Table 1**). Analysis of blood serum samples from women in the maternity ward staff of the same age showed the absence of protective levels of IgG antibodies in a comparable number of cases.

**Table 1:** Comparison of the shares of seronegative to the measles virus persons among the maternity unit employees and women in labor.

Subgroup (age)	Number of seronegative cases (<0,18 IU/ml) in group						Between group^3^
	Employees of the maternity unit			Women in labor			
	n / N^1^	%	95% CI^2^	n / N	%	95% CI	
All women	20 / 88	22.7	14.5–32.9	127 / 594	21.4	18.2–24.9	χ^2^ = 0.1, p = 0.77
21–35 age	10 / 37	27.0	13.8–44.1	117 / 509	23.0	19.4–26.9	χ^2^ = 0.3, p = 0.57
36–43 age	10 / 51	19.6	9.8–33.1	10 / 85	11.8	5.8–20.6	χ^2^ = 1.6, p = 0.21
Between age^3^	χ^2^ = 0.7, p = 0.41			χ^2^ = 5.5, p = 0.02			-

^1^n is the number of seronegative cases, N is the number of women in a group.

^2^the Clopper–Pearson method was used.

^3^the Pearson's chi-squared test was used.

**Figure 1:**
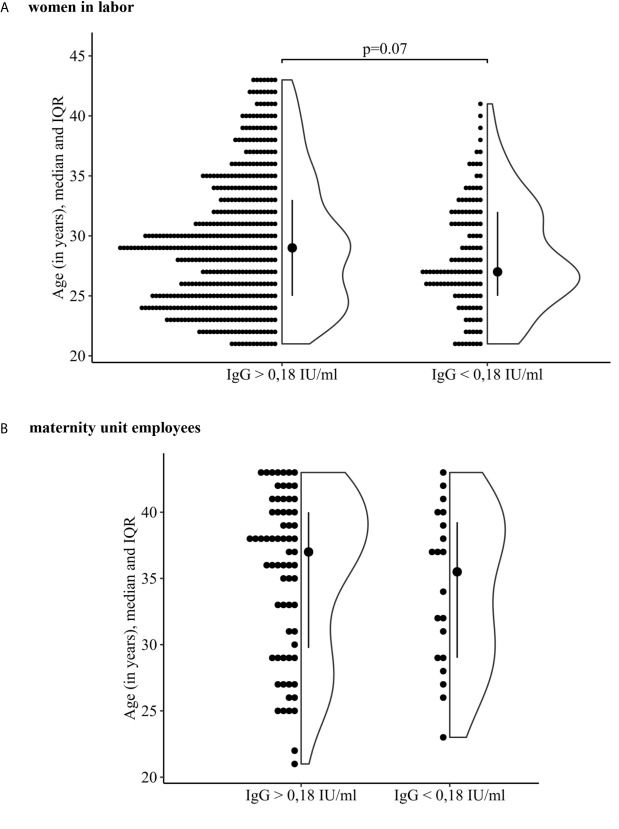
Comparison of the age of women who are seronegative and seropositive to the measles virus. **(A)** women in labor. **(B)** maternity unit employees.

A comparison of the age distributions between seronegative and seropositive to measles virus women in the maternity ward staff and women in labor was made separately (**Figures 1A, B**).

It was revealed that in the group of parturient women, the age distribution of seropositive and seronegative women differed—29 (25, 33) years and 27 (25, 32) years, respectively (p = 0.07 borderline significant). **Figure 1A** shows that among women in labor more were seropositive to the measles virus at an older age compared with those who were seronegative. This is confirmed by the found cut-off point, which was 35 years (Youden’s index was used, J = 0.16, sensitivity 54%, specificity 60%).

Comparison of the proportion of seronegative women in labor in the age subgroups 21–35 years old and 36–43 years old showed that among them, a quarter of the surveyed persons did not have protective antibody levels (23 and 11.8% respectively, p = 0.02) (**Table 1**).

The study of the blood serum of maternity ward staff at the same age showed the absence of a protective antibody level in more than 20% of cases. Among these women, there was also the highest proportion of seronegative persons in the 21–35 year subgroup (27 *vs* 19.6% in 36–46 years subgroup).

However, the percentage of seronegative persons in subgroups of healthy medical personnel of the maternity ward at different ages does not differ significantly between subgroups and when they are compared with similar age subgroups of women in labor.

Thus, the age of women in childbirth less than 35 years is associated with a higher chance to be unprotected against the measles virus infection compared with women in labor who were over 36 years old—OR [95% CI] = 2.2 [1.1–4.5].

Also, women with a low level of IgG to the measles virus (<1.0 IU/ml) have been identified. Analysis of the level of antibodies in seropositive women showed a low level of IgG to the measles virus (<1.0 IU/ml) in 398 samples (2/3 of cases) among women in labor and in 60 samples (68.2%) among the employees of the maternity unit. Thus, in most of the studied serum samples of all women, low values of antibodies were detected (**Table 2**).

**Table 2:** Comparison of the shares of cases with low antibody levels (<1.0 IU/ml) to the measles virus persons among the maternity unit employees and women in labor.

Subgroup (age)	Number of cases with low antibody levels (<1.0 IU/ml)						Between group^3^
	Employees of the maternity unit			Women in labor			
	n/N^1^	%	95% CI^2^	n/N	%	95% CI	
All women	60/88	68.2	57.4–77.7	398/594	67.0	63.1–70.8	χ^2^ =.0.1, p = 0.83
21–35 age	28/37	75.7	58.8–88.2	355/509	69.7	65.5–73.7	χ^2^ = 0.6, p = 0.47
36–43 age	32/51	62.7	48.1–75.9	43/85	50.6	39.5–61.6	χ^2^ = 1.9, p = 0.17
Between age^3^	χ^2 =^ 1,6, p = 0.19			χ^2^ = 12.1, p = 0.001			–

^1^n is the number of cases with low antibody levels, N is the number of women in a group.

^2^the Clopper–Pearson method was used.

^3^the Pearson’s chi-squared test was used.

It was found that in all age subgroups of women in labor, sera with low levels of antibodies predominated (50.6% in 36–43-year old subgroup and 69.7% in 21–35-year old subgroup). Statistically significant differences (p < 0.001) were revealed when comparing the proportions of low-level samples of IgG antibodies in these age subgroups

The age of women in labor less than 35 years old is associated with a higher chance of having a low level of antibodies to measles virus compared with women in labor over 36 years of age—OR [95% CI] = 1.9 [1.2–3.0].

The analysis of the distribution of antibody levels in the serum of maternity ward employees revealed a predominance of low antibody levels in all age subgroups (62.7% in 36–43-year old subgroup and 75.7% in 21–35-year old subgroup). The analysis of low antibody levels did not reveal significant statistical differences between the age subgroups of this group of women.

Samples with high antibody levels (>5.0 IU/ml) were also analyzed. Persons with high levels of antibodies accounted for 7.7% in the group of women in labor, but no person with high level of antibodies was found in the group of employees in the maternity ward. In the group of parturient women, the proportion of women with high levels of antibodies was 7% [4.8–9.4] (35 cases) in the age subgroup of 21–35 years and 13% [6.6–22] (11 cases) in the age subgroup of 36–43 years; the differences are statistically significant (χ^2^ = 3.8, p = 0.05). Among women over 35, the proportion of persons with a high level of antibodies in the group of women in labor is statistically significantly higher than in the group of the maternity ward personnel (p = 0.007 used Fisher’s exact test).

## Discussion

The existing level of population immunity cannot guarantee “herd effect” for this infection in any of the surveyed groups and, subsequently among newborns too. The presence of the combination of these epidemiologically vulnerable three groups creates an extremely unfavorable epidemiological situation in maternity hospitals. Healthcare workers who work in direct contact with patients are susceptible to infectious diseases and can play a role in nosocomial transmission. If they are not protected from controlled infections, they pose a danger to themselves and their patients, especially in an outbreak situation. It is alarming that several studies have reported a lack of immunity against vaccine-preventable infections in healthcare workers (6).

Earlier, a 50–60% decrease in the level of seropositivity towards measles among adolescents and young people under 25 years of age was demonstrated. Surveys of healthcare professionals in 2004 and 2009 also showed a seropositive rate of 78.1% in participants aged 20–29; antibody titers in a doubtful range were detected in 17.1% in this age group (13). Our results confirmed that the level of population immunity is insufficient both among medical workers and among women in labor: the revealed proportion of seronegative persons (about 20%) is more than three times higher than the permissible level necessary to ensure that “herd effect” for this infection is achieved. The largest proportion of seronegative persons was found in the age subgroup of 26–35 years; the proportion of seronegative persons among medical workers (maternity ward employees) and women in labor did not differ significantly from each other.

The global tendency of the incidence of measles to resurge and increase is becoming a serious problem not only because of the suffering and death of patients but also because of the costs of combating measles epidemics which many times exceed the costs of vaccine prevention (14).

In addition, previously, in low-income countries, the use measles vaccine resulted in a 30% reduction in infant mortality, of which only 4% was attributable to deaths directly from measles (15), leading to the perception that exposure to measles vaccine can be much more than just prevention of measles infection. Some features of the measles vaccine are also taking on new and very important importance in the wake of the COVID-19 pandemic.

A 30 amino acid sequence homology has now been established between SARS-Cov-2 Spike (S)-glycoprotein (PDB: 6VSB) and the F1 fusion glycoprotein of measles virus (PDB: 5YXW_B). At the same time, computer analysis of the homologous region made it possible to reveal the antigenic properties of the epitopes located in this area, which explains the similarity of antigenic properties (16, 17).

These data make it possible to explain theoretically and substantiate the protective role of measles immunity against coronavirus infection. In addition, they allow us to understand some clinical and epidemiological observations below. Thus, data obtained in China, Italy, and South Korea show that children under the age of 10 years rarely get sick with COVID-19, and the disease itself occurs most often in a mild and asymptomatic form (18). It is noteworthy that published epidemiological data indicate a correlation between the massive use of anti-measles vaccines and a decrease in mortality among patients with COVID-19 (19).

In addition to cross-specific activity due to the similarity of antigenic determinants of the most important proteins, there is growing evidence that attenuated live vaccines also provide non-specific protection against lethal infections not associated with the target vaccine pathogen by inducing “trained” non-specific innate immune cells that provide increased host resistance to other infections (20).

These are compelling arguments for some authors to consider measles (and some other vaccines) as real supportive protective measures in the fight against the COVID-19 pandemic (21). Thus, today the question of the intensity of the measles immunity, while remaining very relevant in the light of the fight against measles, goes far beyond the scope of this pathology.

The results obtained also indicate that the decrease in the level of post-vaccination measles specific IgG antibodies in the post-elimination period occurs much faster than in previous years when immunological memory was formed as a result of infection with a wild virus (clinically expressed cases of infection or during the formation of a booster immune response in previously vaccinated persons).

It is widely accepted that protective immunity against measles is correlated with levels of neutralizing antibodies, but the actual immunologic determinants of protection are not known. Nevertheless, to our current knowledge, every vaccine measles strain starts the infection process of host cells by interaction of envelope glycoprotein H to virus-specific cell receptors, CD150, which is a signaling lymphocytic activation molecule and CD46, which is an inhibitory complement receptor (22).

That is why some assumptions regarding decreasing immunogenicity of measles vaccines are related to the above-mentioned mechanisms. These are a crucial mutation in the H gene which reduces its interaction with specific cell receptors. As opposed to wild measles strains, the ability of vaccine strains to induce interferon-β production also (23, 24) might influence the immunogenicity of vaccines, but, unfortunately, their role and fine mechanisms are not completely clear yet.

The mentioned aspects are universal for many countries since most vaccines strains are derived from the Edmonstone vaccine strain, including the strains that are used for measles vaccines production in the Russian Federation.

According to the National Schedule of Preventive Vaccinations, children aged 12 months are subject to a single vaccination against measles, rubella, and mumps, followed by revaccination at the age of 6 years. At the same time, additional immunization against measles is required for the following contingents of the population: children aged 1–18 years and adults up to 35 years old, not sick, not vaccinated, vaccinated once, having no information about measles vaccinations; adults 36–55 years old belonging to risk groups (including healthcare workers). For voluntary vaccination in the Russian Federation combination, vaccine formulations against measles are used; the same as the monovalent vaccine. The main characteristics of vaccine preparations registered in the Russian Federation are presented in **Table 3** below.

**Table 3:** Characteristics of the vaccine preparations registered in the Russian Federation for active immunization against measles.

Composition of vaccine	Measles virus strain	Mumps strain	Rubella virus strain	Cell culture
Live virus mumps-measles vaccine (SPA “Microgen”)	Leningrad-16	Leningrad-3	No	Primary quail embryo fibroblast cells
Vactrivir (SPA “Microgen”)	Leningrad-16	Leningrad-3	Wistar RA 27/3	Primary quail embryo fibroblast cells + human diploid cells MRC-5
ММР^®^ II (MSD)	Edmonston	Jeryl Lynn	Wistar RA 27/3	Primary cultures of chick embryo + human diploid cells WI-38
Priorix^®^. (GSK)	Schwarz	RIT 4385 (Jeryl Lynn derived)	Wistar RA 27/3	Primary cultures of chick embryo + human diploid cells MRC-5
Live attenuated vaccine against measles, mumps, and rubella (Serum Institute of India)	Edmonston-Zagreb	Leningrad-Zagreb	Wistar RA 27/3	Primary cultures of chick embryo + human diploid cells
Live virus measles vaccine (cultural, dry) (SPA “Microgen”)	Leningrad-16	No	No	Primary cell culture of quail embryos

So, as a whole, the quality of vaccine preparations and strategy of preventive vaccination in the Russian Federation completely meet WHO recommendations and demands.

Assessment of secondary vaccine ineffectiveness (weakening of immunity or inability to maintain protective immunity over time) can be difficult and requires long-term monitoring of adaptive immunity caused by a measles vaccine after the first and second doses of the vaccine (9).

It seems likely that over time, most persons with low levels of measles antibodies will lose their protective humoral immunity against measles and become vulnerable to infection.

## Conclusions

A high proportion of persons who do not have sufficient protective levels of anti-measles antibodies, which was observed among healthcare employees aged 21–35 (27% of seronegative and 75.7% with low antibody content) and among women in labor (23% of seronegative and 69.7% with low antibody content), can subsequently lead to both nosocomial and out-of-hospital outbreaks of the disease, involving children who have not received protective levels of antibodies from mothers.

Given the new aspects of the importance of measles immunity as a potential protective factor during the COVID-19 pandemic, measures to organize mass immunization against measles not only should not be weakened but also should be strengthened. Under these circumstances, one possible strategy might be the introduction into the National Vaccination Schedule of third anti-measles revaccination or developing a vaccine with greater immunogenicity.

## Data Availability Statement

The raw data supporting the conclusions of this article will be made available by the authors, without undue reservation.

## Ethics Statement

The study was based on the ethical principles and recommendations of the WHO and the Russian Ministry of Health. All patients signed the informed consent for participation in the study before the beginning of the research.

## Author Contributions

MK – the head of the project; PZ – Bibliography Support; LG - Methodology, Resources; KM - Writing - Review and Editing; VP – Investigation; AS – Investigation; VZ - Writing - Review and Editing; DB - Data Curation; DP - Data Curation; AV – Statistic Procedures, Formal analysis; AR– Investigation; EK – Investigation. All authors contributed to the article and approved the submitted version.

## Conflict of Interest

The authors declare that the research was conducted in the absence of any commercial or financial relationships that could be construed as a potential conflict of interest.
